# Clinical Presentation, Course, and Risk Factors Associated with Mortality in a Severe Outbreak of COVID-19 in Rhode Island, USA, April–June 2020

**DOI:** 10.3390/pathogens10010008

**Published:** 2020-12-24

**Authors:** Eleftheria Atalla, Raina Zhang, Fadi Shehadeh, Evangelia K. Mylona, Maria Tsikala-Vafea, Saisanjana Kalagara, Laura Henseler, Philip A. Chan, Eleftherios Mylonakis

**Affiliations:** 1 Infectious Diseases Division, Warren Alpert Medical School of Brown University, Providence, RI 02903, USA; eleftheria_atalla@brown.edu (E.A.); raina_zhang@brown.edu (R.Z.); fadi_shehadeh@brown.edu (F.S.); evangelia_mylona@brown.edu (E.K.M.); maria_tsikala_vafea@brown.edu (M.T.-V.); saisanjana_kalagara@brown.edu (S.K.); philip_chan@brown.edu (P.A.C.); 2 Medicine and Long-Term Care Associates Llc., Cranston, RI 02920, USA; laurahen@cox.net; 3 Rhode Island Department of Health Division of Preparedness, Response, Infectious disease and Emergency Medical Services (PRIDEMS), Providence, RI 02908, USA

**Keywords:** COVID-19, LTCF, nursing homes, pandemic

## Abstract

Long-term care facilities (LTCFs) have had a disproportionally high mortality rate due to COVID-19. We describe a rapidly escalating COVID-19 outbreak among 116 LTCF residents in Rhode Island, USA. Overall, 111 (95.6%) residents tested positive and, of these, 48 (43.2%) died. The most common comorbidities were hypertension (84.7%) and cardiovascular disease (84.7%). A small percentage (9%) of residents were asymptomatic, while 33.3% of residents were pre-symptomatic, with progression to symptoms within a median of three days following the positive test. While typical symptoms of fever (80.2%) and cough (43.2%) were prevalent, shortness of breath (14.4%) was rarely found despite common hypoxemia (95.5%). The majority of patients demonstrated atypical symptoms with the most common being loss of appetite (61.3%), lethargy (42.3%), diarrhea (37.8%), and fatigue (32.4%). Many residents had increased agitation (38.7%) and anxiety (5.4%), potentially due to the restriction measures or the underlying mental illness. The fever curve was characterized by an intermittent low-grade fever, often the first presenting symptom. Mortality was associated with a disease course beginning with a loss of appetite and lethargy, as well as one more often involving fever greater than 38 °C, loss of appetite, altered mental status, diarrhea, and respiratory distress. Interestingly, no differences in age or comorbidities were noted between survivors and non-survivors. Taking demographic factors into account, treatment with anticoagulation was still associated with reduced mortality (adjusted OR 0.16; 95% C.I. 0.06–0.39; *p* < 0.001). Overall, the clinical features of the disease in this population can be subtle and the symptoms are commonly atypical. However, clinical decline among those who did not survive was often rapid with patients expiring within 10 days from disease detection. Further studies are needed to better explain the variability in clinical course of COVID-19 among LTCF residents, specifically the factors affecting mortality, the differences observed in symptom presentation, and rate of clinical decline.

## 1. Introduction

The current pandemic has put long-term care facilities (LTCFs) into crisis, with the high mortality among residents challenging the health care infrastructure [1]. In the US, less than 1% of the population lives in LTCFs, and yet this small fraction of the country accounts for approximately half of COVID-19 related mortality [2]. 

Compared to community-dwelling older individuals, the elderly who reside in LTCFs are at higher risk for infection and mortality from SARS-CoV-2 [3], given the congregate care setting and population of individuals with cognitive impairment, challenging infection control measures [3]. The importance of masking, physical distancing, environmental cleaning, hand hygiene, and ventilation, is widely emphasized for infection control in confined spaces [4]. Outbreaks in LTCFs are associated with inadequate ventilation systems increasing the risk of airborne SARS-CoV-2 transmission [5], while significant shortages in testing supplies and personal protective equipment (PPE), as well as staffing constraints, have further exacerbated the detrimental impact of COVID-19 on this population [6,7].

In addition to the infection control recommendations for LTCFs issued by the Centers for Medicare and Medicaid Services (CMS) in consultation with the Centers for Disease Control and Prevention (CDC) [8], insight into the clinical presentation of these patients is important for prompt identification of infected cases, subsequent isolation and quarantine measures, and improvement of clinical outcomes. Existing studies on COVID-19 outbreaks in the institutionalized elderly largely focus on epidemiological characteristics [9,10,11,12] and clinical symptoms related to initial presentation and transmission [13,14,15,16,17,18]. Few studies assessed the clinical course and past initial symptom status of residents, as it relates to mortality outcomes [19,20,21], especially in a non-hospital setting [22,23], with an infection and mortality rate as high as those documented here. In this study, we report a COVID-19 outbreak among 116 residents of a nursing home with a very high infection and mortality rates, with a specific focus on the clinical presentation and characteristics associated with mortality.

## 2. Materials and Methods 

### 2.1. Patient Population and Data Collection

We retrospectively collected data from residents’ electronic health records from 14 April to 15 June 2020 in Rhode Island, USA. The 145-bed facility had two floors accommodating patients requiring skilled nursing and rehabilitation care since 1999. On 14 April 2020, the facility reported a widespread COVID-19 outbreak. At the time of the COVID-19 outbreak, the facility census was 116 residents.

Variables collected included—demographics (age, gender, race), comorbidities, clinical symptoms, radiographic imaging results, vitals (temperature, oxygen saturation), medical interventions such as supplementary oxygen and anticoagulation, laboratory measurements (inflammatory markers, CBC, etc.), and outcomes (death, hospital admission).

### 2.2. SARS-CoV-2 Identification 

As part of the outbreak response, samples were collected from nasopharyngeal SARS-CoV-2 swabs and were sent to the laboratories of the RI Department of Health. The standard technique used to identify SARS-CoV-2 was real-time reverse-transcriptase polymerase chain reaction (RT-PCR). The molecular assays used were GeneXpert^®^ Systems (Cepheid, Sunnyvale, CA, USA) and Aptima^®^ Systems (Hologic Inc., San Diego, CA, USA). The turnaround time for the COVID-19 molecular diagnostic testing was 48 h. 

### 2.3. Statistical Analysis 

Chi-squared tests were used to compare categorical variables, and Wilcoxon rank sum tests were used for continuous variables. We performed a logistic regression analysis in order to identify the impact of anticoagulation on mortality. Age, gender, race, and comorbidities (using the Weighted Elixhauser Score) were included in the logistic regression model to account for confounding factors. Statistical analyses were performed with the Stata statistical software (Version 15; StataCorp, College Station, TX, USA). The statistically significant threshold was set at 0.05.

### 2.4. Ethical Approval 

The study was determined exempt by the Institutional Review Board of Rhode Island Hospital (#412820) and, given the retrospective nature of the investigation, informed consent was waived.

## 3. Results

The first laboratory-confirmed case within the facility was an asymptomatic staff member who was identified on April 8th. Based on guidance from the RI Department of Health, the first testing round was initiated on April 14th and included only residents with whom the staff member potentially interacted. A second round of testing was immediately performed after the first round of tests results came back positive.

Prior to the outbreak, on 12 March 2020, the facility implemented mitigation measures, including closing the site to visitors, curtailment of new admissions, canceling community dining and group activities, and universally using surgical masks. The use of N95 masks was initiated on 14 April 2020 when the first resident tested positive and full personal protective equipment (PPE) was used only in the COVID-19 positive wings or during interaction with symptomatic residents, regardless of the test result. The last testing round occurred on 6 June 2020. The outbreak was considered resolved in mid-June 2020, as no new COVID-19 cases were detected for two consecutive weeks and a majority of the residents had a clinical resolution of symptoms.

Of the 116 residents in the facility, 111 tested positive for SARS-CoV-2 within the specified time period, demonstrating an overall infection rate of 95.6%. Demographic and clinical characteristics of individuals with confirmed COVID-19 are included in Table 1. Median age of patients was 87 years (IQR 77.00–92.00), with female (79.3%) and Caucasian (97.3%) predominance. The most prevalent comorbidities were hypertension (84.7%) and cardiovascular disease (84.7%), followed by dementia (68.5%), renal disease (35.1%), diabetes (34.2%), and chronic pulmonary disease (29.7%). Obesity was rarely found (7.2%) (Table 1). 

The most common symptoms throughout the whole illness course were fever (80.2%), particularly low-grade fever (37.0–37.9 °C) (64.9%), loss of appetite (61.3%), altered mental status (59.5%) with lethargy (42.3%), and new onset or worsening cough (43.2%). Other symptoms observed included diarrhea (37.8%), fatigue (32.4%), and respiratory distress (23.4%). In particular, low grade fever (32.4%) and loss of appetite (17.1%) (Table 1) were commonly the presenting symptoms. 

Notably, a considerable proportion of residents (33.3%) were asymptomatic at the time of testing and instead developed symptoms after a median of 3 days (IQR 1–5), following the positive test, whereas 9% of patients were asymptomatic throughout the whole illness course.

Laboratory workup was performed in 81% (90/111) of the COVID-19 positive residents. The residents who did not have a laboratory workup (21/111) were asymptomatic patients, hospice care patients, or residents whose proxies declined labs. Tests performed included complete blood count (CBC) (83/90), liver function tests (LFTs) (80/90), D-dimers (68/90), and inflammatory markers such as procalcitonin (48/90), ferritin (16/90), and CRP (10/90). All abnormal values documented in the data were from labs taken within 10 days of the first positive test of that resident and before the first negative test, indicating the post-infectious period. Some of the most common laboratory abnormalities included elevated D-dimers (>0.51 μg/mL) (79%), low hemoglobin (57%), and increased ferritin (>370 ng/mL in males and >200 ng/mL in females) (56%), CRP (>5 mg/L) (40%), and procalcitonin (>0.15 ng/ml) (10%). Thrombocytopenia (22%) and leukopenia (16%) were also commonly reported (Table 1).

Of the 111 residents, 47 had a chest radiograph, of which 27 (57.4%) were abnormal. The most common findings on radiograph were interstitial densities (8 patients [29.6%]), unilateral base infiltrates (7 [29.6%]), patchy unilateral densities (5 [18.5%]), and patchy bilateral densities (4 [14.8%]).

### Mortality Outcomes and Treatment 

Overall, the outbreak caused a significant mortality rate of 43.2%, with a median of 10.5 days (IQR 7.50–18.50) from symptom onset to death. The peak of deaths was observed between April 30^th^ and May 6^th^, when 43.2% of the total deaths occurred.

Interestingly, there was a more striking symptomatology in the deceased, compared to the patients who survived. Particularly, the disease course was more likely to begin with a loss of appetite (31.3% vs. 6.3%, *p* < 0.001) and lethargy (10.4% vs. 1.6%, *p* = 0.042) in patients who expired than in those who survived. Additionally, during their clinical course, the deceased patients had a higher incidence of fever greater than 38 °C (64.6% vs. 42.9%, *p* = 0.023), loss of appetite (83.3% vs. 44.4%, *p* < 0.001), altered mental status (85.4% vs. 39.7%, *p* < 0.001), diarrhea (50% vs. 28.6%, *p* = 0.021), and respiratory distress (52.1% vs. 1.6%, *p* < 0.001). Patients who survived were more commonly asymptomatic and suffered from headaches, more frequently than the deceased patients [(15.9% vs. 0%, *p* = 0.004) and (9.5% vs. 0%, *p* = 0.028), respectively].

Deceased patients also experienced severe hypoxemia (SpO2 < 75%) more often than those who survived (16.7% vs. 4.8%, *p* = 0.04) and subsequently received supplemental oxygen more frequently (66.7% vs. 27%, *p* < 0.001). Laboratory work-up revealed a higher incidence of leukocytosis (17% vs. 4%, *p* = 0.034), and elevated procalcitonin (24% vs. 3%, *p* = 0.028) among the deceased, whereas leukopenia was more commonly observed in patients who survived (22% vs. 3%, *p* = 0.025) (Table 1). No significant differences in demographic characteristics and comorbidities were noted between the patients who survived and those who did not.

Most of the residents had Do-Not-Resuscitate (DNR) and Do-Not-Intubate (DNI) orders. After discussion with families or powers of attorney (POA), most residents were treated in the facility for the entirety of their illness. The vast majority of families declined hospital transfer and opted for treatment that was available at the facility. Treatment in place included supplemental oxygen, fluids and antibiotics when indicated, and comfort palliative medications for residents who were nearing end of life. Therefore, hospital admission rate was low (1.8%).

In addition, 63.1% (70/111) residents received anticoagulation at the time of the outbreak. Anticoagulation was not initiated in residents in the following situations—those that had none to mild symptoms, those that were on hospice or that refused treatment, those that were already on anticoagulants, and those that had contraindications (including a history of significant bleeding, hepatic injury, and relative thrombocytopenia). Survivors received anticoagulation more often (79.4%) compared to non-survivors (41.7%) and that difference was statistically significant (*p* < 0.001). Thus, residents receiving anticoagulation were 84% more likely to survive compared to residents not receiving anticoagulation.

Specifically, prophylactic anticoagulation with enoxaparin was initiated in 47.7% (53/111) patients, whereas 15.3% (17/111) were already under chronic anticoagulation with oral anticoagulants (see Table 1 for anticoagulant breakdown). When we examined each anticoagulant separately, survivors received enoxaparin more often compared to non-survivors (65.1% vs. 25.0%, *p* < 0.001), while no significant differences were observed between the survivors and deceased patients receiving rivaroxaban (3.2% vs. 8.3%, *p* = 0.23), apixaban (6.3% vs. 2.1%, *p* = 0.28), or coumadin (4.8% vs. 4.2%, *p* = 0.88).

The logistic regression analysis showed that anticoagulation was associated with reduced mortality (adjusted OR 0.16; 95% C.I. 0.06–0.39; *p* < 0.001), even after adjusting for age, gender, race, and comorbidities. The analysis showed that this association between survival and anticoagulation therapy remained significant for residents receiving enoxaparin (adjusted OR 0.14; 95% C.I. 0.05–0.35; *p* < 0.001). No statistically significant association was observed in residents receiving coumadin (adjusted OR 0.82; 95% C.I. 0.12–5.48; *p* = 0.840), apixaban (adjusted OR 0.33; 95% C.I. 0.03–3.28; *p* = 0.347), or rivaroxaban (adjusted OR 3.01; 95% C.I. 0.50–18.12; *p* = 0.228).

## 4. Discussion

Our study describes a COVID-19 outbreak among 116 LTCF residents in Rhode Island, USA, lasting for a period of two months during the initial wave of the pandemic that challenged the health resources. Despite timely stringent mitigation, a high infection rate (95.6%) was observed, with more than half of the residents contracting the disease within 19 days from the first confirmed case in the facility. We also found a high mortality rate (43.2%), with fatalities occurring in a median of 10.5 days from symptom onset. Although fever can be absent or blunted in the elderly [24], in our cohort the most common presenting symptom was fever, followed by cough and atypical COVID-19 manifestations. Particularly, among patients with a fatal outcome, the disease was more likely to start with a loss of appetite and altered mental status, compared to those who survived. 

We also observed a significant percentage (34.2%) of patients who were asymptomatic at the time of testing but exhibited symptoms later in the infection course, and a smaller proportion of completely asymptomatic (8.1%) patients. Previous studies on COVID-19 in nursing homes reported similar findings [13,15], underscoring the contribution of asymptomatic carriers to disease transmission [25,26]. 

Reinforcing findings in recent reports [13,16], we found a high prevalence of atypical symptoms, of which loss of appetite and symptoms of encephalopathy (lethargy, confusion) were the most common. In addition to a hypoactive mental state, increased anxiety and agitation were also observed, especially among residents with a pre-existing cognitive dysfunction. Indeed, extended periods of social isolation can have detrimental effects on the mental health of the elderly, causing emotional distress and further cognitive decline [27]. Additionally, despite the presence of hypoxemia, shortness of breath was rarely found, except in those who had a severe respiratory disease. However, dyspnea, especially dyspnea upon exertion, was difficult to evaluate given the limited ambulatory ability of many residents and the restricted movement inside the facility, due to the infection control measures. Fever also followed a specific pattern in these patients. Intermittent low-grade fever was often the earliest symptom of the disease, with occasional spikes occurring during the illness course.

The data from this cohort also suggest variability in clinical course between patients who survived and those who did not, despite non-significant differences in demographics and comorbidities. Compared to the survival group, infection in those who expired was more likely to begin with lethargy and loss of appetite, symptoms that persisted during the whole illness course. Clinical decline was often sudden and unexplained, with deaths occurring soon after the initial symptoms. Importantly, prevalence of initial atypical symptoms had serious implications for clinical decision making, specifically in early consideration of COVID-19 testing and application of the infection protocol, given the rapid clinical decline witnessed in the deceased group.

Elevated inflammatory markers (such as leukocytosis and high procalcitonin) were also more common among residents with a poor outcome. These findings might indicate a concurrent or superimposed bacterial infection, which could contribute to clinical deterioration of these patients. Indeed, Zhou et al. showed that half of the hospitalized COVID-19 patients who died had a superimposed bacterial infection [28].

With regards to treatment, prophylactic anticoagulation therapy was significantly associated with reduced mortality. In light of reports describing coagulopathy in severe COVID-19 disease, prophylactic anticoagulation therapy was given to 63.1% residents (70/111 residents), on average 9.5 days after their first positive test. Residents receiving prophylactic anticoagulation were 84% less likely to die compared to those who did not take any anticoagulation. Due to the small numbers taking anticoagulation other than enoxaparin, conclusions regarding differences in the impact of specific anticoagulants could not be made. Current evidence for use of prophylactic anticoagulation in COVID-19 patients is predominantly based on observational studies of inpatient populations [29], though incidence of pulmonary embolism even in non-hospitalized asymptomatic COVID-19 patients without other predisposing factors for thrombosis suggests that even outpatients could potentially benefit from anticoagulation [30,31]. We found only one anticoagulation study in the LTCF population, a retrospective multi-center case series of 101 Dutch nursing home residents, that reported no significant evidence of mortality benefit with use of oral antithrombotic therapy [32]. Further studies are needed to characterize the benefit of prophylactic anticoagulation in outpatient settings, like LTCFs.

While trends in clinical presentation might be better visualized in the context of widespread infection and high mortality, the reasons behind the increased infection rate are less clear cut. It is interesting to note that the first confirmed case of COVID-19 in the facility was in an asymptomatic staff member. While the facility itself was able to implement strict mitigation policies, including restriction of visitors, staff were routinely coming in from the community. Furthermore, staff members might be more likely to present as asymptomatic cases than nursing home residents [13,33,34]. While analysis of staff transmission was beyond the scope of this study, staff infection was identified as a strong risk factor for resident mortality, alongside a temporal relationship that suggests residents were infected by staff [35]. Specific nursing home characteristics might factor into the infection rate as well. Poorly ventilated buildings were implicated in a study of a COVID-19 outbreak in a Dutch nursing home [5,36]. Studies of nursing home quality metrics also found a relationship between lower Center for Medicare and Medicaid Services’ (CMS) star quality ratings, specifically staffing deficiencies and for-profit ownership status of nursing homes, and COVID-19 outbreak risk and severity [37,38,39,40].

The clinical characteristics observed in this LTCF cohort are notable in the context of the large cluster of infected and high mortality rate, among the highest reported in current literature to our knowledge [14]. Incidence, demographic associations [20], transmission and symptom status [9,10,11,12,13,15,16,19], and atypical presentation [17,18,21] in LTCF residents are widely discussed in the literature [14]. Less attention was paid to the clinical patterns of COVID-19 in LTCF residents, as it relates to mortality outcomes, and those that did were not in exclusively outpatient settings [22,23]. The unique combination of a congregate outpatient setting, staff interaction, and resident characteristics suggests differences in exposure, illness course, and risk of sequelae like venous thromboembolisms, when compared to hospitalized or community-dwelling patients [35]. Remdesivir, currently the only FDA-approved treatment for COVID-19, is not an option in outpatient settings. Characterization of the clinical picture relating to the outcomes in the outpatient LTCF population is important for the discussion of treatment guidelines for this health care setting. Ongoing efforts by the scientific community should be made to better address treatment options for COVID-19 in LTCF residents, in light of the morbidity and mortality burden upon this specific population.

Our analysis presents some limitations. First, the study has inherent biases associated with its methodology (retrospective design, small sample size). Second, imaging and laboratory tests were not performed universally and therefore might yield skewed results. Additionally, given the increased workload during the pandemic, the possibility exists that some signs and symptoms were under-reported into the electronic health record system. Finally, due to the low number of negative cases in the facility, a risk factor analysis for infection among residents was not feasible.

In summary, we report a COVID-19 outbreak that caused a considerably high infection and fatality rate among the residents of a long-term care facility. Clinical presentation often included, and more significantly, began with atypical symptoms such as loss of appetite and altered mental status with notable lethargy, especially among those who died. Despite their more severe disease course, patients who expired did not have any significant differences in age or comorbidities, compared to those who survived. Notably, lower mortality was seen in residents taking prophylactic anticoagulation. Further research is warranted to investigate the reasons behind variations in clinical outcomes and use of anticoagulation among this population.

## Figures and Tables

**Table 1:** Demographic, Clinical Characteristics, and Laboratory Findings of Residents with Confirmed COVID-19.

	Total	Alive	Deceased	*p*-Value
	N = 111	N = 63	N = 48	
Age (median [IQR])	87.0 (77.0–92.0)	85.0 (73.0–92.0)	88.0 (79.0–92.0)	0.32
<65	5 (4.5%)	3 (4.8%)	2 (4.2%)	
65–84	43 (38.7%)	27 (42.9%)	16 (33.3%)	
≥85	63 (56.8%)	33 (52.4%)	30 (62.5%)	
Sex				0.65
F	88 (79.3%)	49 (77.8%)	39 (81.3%)	
M	23 (20.7%)	14 (22.2%)	9 (18.8%)	
Ethnicity				0.50
Caucasian	108 (97.3%)	62 (98.4%)	46 (95.8%)	
Asian	1 (0.9%)	0 (0.0%)	1 (2.1%)	
Black	2 (1.8%)	1 (1.6%)	1 (2.1%)	
Chronic underlying conditions				
Hypertension	94 (84.7%)	52 (82.5%)	42 (87.5%)	0.47
Cardiovascular disease	94 (84.7%)	50 (79.4%)	44 (91.7%)	0.075
Alzheimer’s disease or other dementias	76 (68.5%)	39 (61.9%)	37 (77.1%)	0.088
Renal disease	39 (35.1%)	19 (30.2%)	20 (41.7%)	0.21
Diabetes	38 (34.2%)	23 (36.5%)	15 (31.3%)	0.56
Chronic pulmonary disease	33 (29.7%)	20 (31.7%)	13 (27.1%)	0.59
History of malignancy	7 (6.3%)	2 (3.2%)	5 (10.4%)	0.12
Obesity	8 (7.2%)	5 (7.9%)	3 (6.3%)	0.73
Liver disease	1 (0.9%)	1 (1.6%)	0 (0.0%)	0.38
Weighted Elixhauser Score				0.56
<0	20 (18.0%)	13 (20.6%)	7 (14.6%)	
0	3 (2.7%)	1 (1.6%)	2 (4.2%)	
1–4	23 (20.7%)	11 (17.5%)	12 (25.0%)	
≥5	65 (58.6%)	38 (60.3%)	27 (56.3%)	
Initial presenting symptoms				
Fever				
T 37–37.9 °C (98.7–100.3 F)	36 (32.4%)	21 (33.3%)	15 (31.3%)	0.82
T > 38 °C (100.4 F)	24 (21.6%)	14 (22.2%)	10 (20.8%)	0.86
Loss of appetite	19 (17.1%)	4 (6.3%)	15 (31.3%)	<0.001
Cough	17 (15.3%)	9 (14.3%)	8 (16.7%)	0.73
Diarrhea	8 (7.2%)	4 (6.3%)	4 (8.3%)	0.69
Fatigue	7 (6.3%)	2 (3.2%)	5 (10.4%)	0.12
Lethargy	6 (5.4%)	1 (1.6%)	5 (10.4%)	0.042
Shortness of breath	4 (3.6%)	2 (3.2%)	2 (4.2%)	0.78
Anxiety	3 (2.7%)	2 (3.2%)	1 (2.1%)	0.73
Nausea/vomit	2 (1.8%)	0 (0.0%)	2 (4.2%)	0.10
Respiratory distress	1 (0.9%)	1 (1.6%)	0 (0.0%)	0.38
Rhinorrhea	1 (0.9%)	0 (0.0%)	1 (2.1%)	0.24
Symptoms (at any point in illness)				
Asymptomatic	10 (9.0%)	10 (15.9%)	0 (0.0%)	0.004
Pre-symptomatic	37 (33.3%)	20 (31.7%)	17 (35.4%)	
Time to symptom onset (median [IQR])	3.00 (1.00–5.00)	4.00 (2.00–8.00)	1.00 (1.00–3.00)	0.004
Fever	89 (80.2%)	46 (73.0%)	43 (89.6%)	0.030
T 37–37.9 °C (98.7–100.3 F)	72 (64.9%)	41 (65.1%)	31 (64.6%)	0.96
T > 38 °C (100.4 F)	58 (52.3%)	27 (42.9%)	31 (64.6%)	0.023
Loss of appetite	68 (61.3%)	28 (44.4%)	40 (83.3%)	<0.001
Altered mental status	66 (59.5%)	25 (39.7%)	41 (85.4%)	<0.001
Lethargy	47 (42.3%)	14 (22.2%)	33 (68.8%)	<0.001
Agitation/restlessness	43 (38.7%)	13 (20.6%)	30 (62.5%)	<0.001
Confusion	16 (14.4%)	8 (12.7%)	8 (16.7%)	0.56
Cough	48 (43.2%)	25 (39.7%)	23 (47.9%)	0.39
Diarrhea	42 (37.8%)	18 (28.6%)	24 (50.0%)	0.021
Fatigue	36 (32.4%)	19 (30.2%)	17 (35.4%)	0.56
Respiratory distress	26 (23.4%)	1 (1.6%)	25 (52.1%)	<0.001
Shortness of breath	16 (14.4%)	6 (9.5%)	10 (20.8%)	0.093
Myalgia	14 (12.6%)	6 (9.5%)	8 (16.7%)	0.26
Nasal congestion/rhinorrhea	7 (6.3%)	4 (6.3%)	3 (6.3%)	0.98
Sore throat	6 (5.4%)	5 (7.9%)	1 (2.1%)	0.18
Anxiety	6 (5.4%)	5 (7.9%)	1 (2.1%)	0.18
Headache	6 (5.4%)	6 (9.5%)	0 (0.0%)	0.028
Supplemental Oxygen or Increased Oxygen Requirements	49 (44.1%)	17 (27.0%)	32 (66.7%)	<0.001
Hypoxemia (SpO2 <94% RA)	106 (95.5%)	60 (95.2%)	46 (95.8%)	
Mild hypoxemia (*SpO2* 90–94% RA)	91 (82.7%)	54 (87.1%)	37 (77.1%)	0.17
Moderate hypoxemia (*SpO2* 75–89% RA)	48 (43.6%)	15 (24.2%)	33 (68.8%)	<0.001
Severe hypoxemia (*SpO2* <75% RA)	11 (10.0%)	3 (4.8%)	8 (16.7%)	0.040
Abnormal Chest X-Ray	27 (57%)	11 (46%)	16 (70%)	0.100
Labs *				
Low Hb (<12 g/dL)	47 (57%)	32 (59%)	15 (54%)	0.62
Low platelet count (<150 k)	18 (22%)	9 (16%)	9 (32%)	0.099
High platelet count (>400 k)	9 (11%)	6 (11%)	3 (11%)	0.96
Low WBC count (<4 k/μL)	13 (16%)	12 (22%)	1 (3%)	0.025
High WBC count (>10 k/μL)	7 (8%)	2 (4%)	5 (17%)	0.034
Low neutrophil count (<2 k/μL)	13 (16%)	11 (20%)	2 (7%)	0.13
High neutrophil count (>8 k/μL)	4 (5%)	1 (2%)	3 (11%)	0.074
Low lymphocyte count (<0.8 k/μL)	3 (4%)	2 (4%)	1 (4%)	0.99
High lymphocyte count (>4 k/μL)	0 (0%)	0 (0%)	0 (0%)	
Elevated AST (>40 U/L)	13 (16%)	8 (14%)	5 (19%)	0.57
Elevated ALT (>40 U/L)	3 (4%)	2 (4%)	1 (4%)	0.95
Elevated ALP (>146 U/L)	4 (5%)	4 (7%)	0 (0%)	0.16
Elevated d-dimers (>0.51 μg/mL)	54 (79%)	42 (81%)	12 (75%)	0.62
Elevated CRP (≥5 mg/L)	4 (40%)	2 (25%)	2 (100%)	0.053
Elevated PCT (>0.15 ng/mL)	5 (10%)	1 (3%)	4 (24%)	0.028
Elevated Ferritin	9 (56%)	4 (50%)	5 (63%)	0.61
Treatment				
Anticoagulation	70 (63.1%)	50 (79.4%)	20 (41.7%)	<0.001
Enoxaparin	53 (47.7%)	41 (65.1%)	12 (25.0%)	<0.001
Rivaroxaban	6 (5.4%)	2 (3.2%)	4 (8.3%)	0.23
Apixaban	5 (4.5%)	4 (6.3%)	1 (2.1%)	0.28
Coumadin	5 (4.5%)	3 (4.8%)	2 (4.2%)	0.88
Dabigatran	1 (0.9%)	0 (0.0%)	1 (2.1%)	0.25

ALP, alkaline phosphatase; ALT, alanine aminotransferase; AST, aspartate aminotransferase; CRP, C-Reactive Protein; CXR, Chest X-Ray; PCT, Procalcitonin; RA, Room Air; and WBCs, White Blood Cells. * As lab tests were not ordered for all residents, the percentages reported are calculated from the number of residents that underwent the specified test. Depending on the column, the denominator was either the total number COVID+ residents tested, number COVID+ residents tested who survived, or number COVID+ residents tested who died.

## Data Availability

The data presented in this study are available on request from the corresponding author.
